# Purification, Characterization and Mechanistic Evaluation of Angiotensin Converting Enzyme Inhibitory Peptides Derived from *Zizyphus Jujuba* Fruit

**DOI:** 10.1038/s41598-020-60972-w

**Published:** 2020-03-04

**Authors:** Mina Memarpoor-Yazdi, Hadi Zare-Zardini, Navid Mogharrab, Leila Navapour

**Affiliations:** 1 Department of Chemistry, College of Sciences, Shiraz University, Shiraz, Iran; 2 Hematology and Oncology Research Center, Shahid Sadoughi University of Medical Sciences, Yazd, Iran; 3 Biophysics and Computational Biology Laboratory (BCBL), Department of Biology, College of Sciences, Shiraz University, Shiraz, Iran

**Keywords:** Enzyme mechanisms, Enzymes, Structural biology, Biomaterials - proteins, Biochemistry, Biophysics, Biotechnology, Chemical biology, Computational biology and bioinformatics, Drug discovery, Diseases

## Abstract

The synthetic Angiotensin Converting Enzyme (ACE) inhibitors have side effects and hence demands for natural ACE inhibitors have been rising. The aim of this study is to purify and introduce natural ACE inhibitors extracted from *Zizyphus jujuba* fruits. Proteins from *Zizyphus jujuba* were lysed by trypsin, papain and their combination. Acquired peptides were purified and evaluated for ACE inhibitory activity. Peptide fractions with inhibitory activity were sequenced using tandem mass spectrometry. To elucidate the mode of peptide binding to ACE, homology modeling, molecular docking and molecular dynamics simulation were performed. Amino acid sequence of F2 and F4 peptides, which were the most active hydrolysates, were determined to be IER and IGK with the IC_50_ values of 0.060 and 0.072 mg/ml, respectively. Results obtained by computational analysis revealed that similar to the common ACE competitive inhibitors such as captopril, IER tripeptide binds to the enzyme active site, in vicinity of the zinc binding site, and occupies the S1 and S2’ subsites. Binding occurs through hydrogen bonding with Gln293, Lys522, His524, Tyr531 and also several hydrophobic interactions. Collectively, these findings indicate that IER tripeptide inhibits the rabbit ACE enzyme through a competitive mechanism of inhibition with IC_50_ values in the millimolar range.

## Introduction

Angiotensin converting enzyme (ACE) inhibitors are a new class of drugs which are critical in the treatment of hypertension and heart failure^1^. Although the synthetic drugs are often prescribed due to their well-known role and convenience of access, they have undesirable effects, such as extreme low blood pressure, cough, bad tastes and allergic reactions. Contrariwise, natural inhibitors regulate hypertension under mild conditions without significant side effects; hence, they can be considered as worthy alternatives to synthetic drugs^2^.

ACE inhibitory peptides are the best known amongst various classes of regulatory bioactive peptides due to the development of functional foods with homeostasis role in the human body^3^. ACE is a Zn-metallopeptidase that removes a dipeptide from the decapeptide angiotensin-I to produce the potent vasoconstriction octapeptide angiotensin-II^4^. Several peptides originated from animal or plant proteins exhibit significant therapeutic/regulatory performances like antihypertensive, antimicrobial, antioxidant, immunoregulatory, anticancer activities, etc^3^.

Investigations to find more bioactive peptides, especially multifunctional ones have attracted the attention of researchers. The bioactive peptides are often inactive in their natural forms and when released by enzymatic hydrolysis, display their biological activity^3,5^. So far, several ACE inhibitory peptides have been reported from hydrolysis of different food proteins, such as hen egg white lysozyme^4^, fish^6^, corn^7^, soybean^8^, milk^9^, casein^10^, etc. These peptides are good candidates to regulate blood pressure and also to balance fluid and electrolytes in mammals.

*Zizyphus jujuba* is a well-known medicinal plant with several bioactivities, such as antimicrobial^11^, antidiabetic^12,13^, hypoglycemic^13^, and analgesic^14^. Likewise, we recently reported some antioxidant peptides extracted from *Z. jujuba*^5^. Although there are several reports on the presence of different bioactive compounds in this plant, there is no evidence indicating the presence of ACE inhibitory peptides. In the present study, from protein hydrolysate of *Z*. *jujuba* fruit, two ACE inhibitory peptides were purified and characterized. Investigating the inhibition pattern of these peptides showed that they inhibited the enzyme action by competitive mechanism. As a complement to our experiment, homology modeling, molecular docking and 100 nanoseconds (ns) molecular dynamics (MD) simulation were performed to provide an atomistic insight into the interaction of IER peptide (due to the higher affinity) with the ACE.

## Materials and Methods

### Materials

TFA (trifluoroacetic acid), acetonitrile, trypsin (from bovine pancreas), papain (from pawpaw sap), FAPGG (N-(3-[2-furylacryloyl-Phe-Gly-Gly])), ACE (angiotensin-I converting enzyme) from rabbit lung and captopril (≥98% purity), were purchased from Sigma-Aldrich Co. (Saint Louis, MO, USA). Ultrafiltration membranes with a 3 kDa cut-off were purchased from Millipore (Bedford, MA, USA). Analytical and semi-preparative columns were purchased from Macherey Nagel GmbH Co. (St. Neumann Neander, Düren, Germany). All other chemicals used were of analytical grades.

### Protein extraction and hydrolysate preparation

Proteins were extracted from *Z. jujuba* fruit according to the method described by Memarpoor-Yazdi *et al*.^5^. Briefly, *jujuba* fruits were soaked in water and then crushed in a porcelain mortar to attain a homogeneous semi-solid mixture. Excess salt was removed by centrifugation (5000 g, 10 min) and the supernatant was filtered using a filter paper. The extract from *jujuba* fruit was brought from zero to 85% concentration using the standard ammonium sulphate precipitation procedure, and then saturated with salt using gentle agitation at 4 °C. Extracted proteins were suspended in phosphate buffer (50 mM, pH 7.8) forming colloidal particles. Deposits including small and large proteins in the solution were collected by centrifugation (5000 g, 20 min), dissolved in distilled deionized H_2_O (ddH_2_O) and dialyzed against water at 4 °C for 24 hours. Samples separated by dialysis were lyophilized and their proteins were quantified by Bradford method^15^, using bovine serum albumin as the control. A total of 500 mg lyophilized powder was prepared from 10 g *jujuba* fruit. After ammonium sulphate precipitation, the fruit powder yielded 6 mg protein.

Enzymatic hydrolysis is essential for releasing bioactive peptides. Accordingly, the extracted proteins were incubated with two commercial enzymes, papain and trypsin, and a combination of the two. Using these enzymes and a protein/enzyme ratio of 50:1, the protein solution (4 mg/ml) in Tris-HCl buffer (50 mM, pH 7.5) was hydrolyzed. Each enzyme was dissolved separately (0.08 mg/ml) in the same buffer. The hydrolysis reaction was conducted at 37 °C for 4 h. Finally, hydrolysis reaction was stopped by heating the solution in boiling water for 15 min. The hydrolysates were centrifuged at 7000 g for 10 min and the supernatants were collected and transferred to test tubes for further investigations.

### Peptides purification and enzymatic assay

The prepared hydrolysates were filtered using a 3 kDa cut-off ultramembrane and subsequently were subjected to peptide purification using a C_18_ semi-preparative RP-HPLC column (10 × 250 mm, supplied by Macherey-Nagel GmbH & Co., Düren, Germany). Elution was performed using 0.1% trifluoroacetic acid (TFA) in ddH_2_O (v/v) as solution A and a 50% gradient of 0.098% TFA in acetonitrile as solution B for 55 min at 1 ml/min flow rate. Finally, the absorbance of the eluted peaks was recorded at 220 nm and major peaks were lyophilized and subjected to enzymatic assay. Among active fractions, two fractions with the maximum ACE inhibitory potential were purified more using the same RP-HPLC column with a gradient of 0.5% eluent B/min.

Activity of the peptide fractions for ACE inhibition was measured according to our previous study^16^. Samples contained hydrolysate or peptide (50 µl, 1 mg/ml), ACE (22 µl, 50 mU/ml), FAPGG (100 µl, 0.5 mM) and ACE buffer (150 µl, 50 mM Tris-HCl pH 7.5, 0.3 M NaCl and 1 mM ZnCl_2_). The control was the same as the samples, except that ACE buffer (50 µl) was used instead of inhibitor sample. Absorbance changes were measured for 30 min at 340 nm. ACE inhibition percentage was obtained based on the following equation:

$${\rm{ACE}}\,{\rm{inhibition}}( \% )=[1-({\Delta {\rm{A}}}_{{\rm{inhibitor}}}/{\Delta {\rm{A}}}_{{\rm{control}}})]\times 100$$

Peptide concentration to inhibit 50% ACE activity (defined as IC_50_) was calculated by plotting the ACE inhibition percentage against different peptide concentrations. Experiments were performed in triplicates. The IC_50_ values of the purified peptides were compared with that reported for captopril (used as a positive control).

### Determination of amino acid sequence and the inhibition pattern

After further purification, the most active peptides, F2 and F4, were selected to determine their amino acid sequences. Desalting of the samples was carried out using ZipTips [Millipore]. Desalted peptides were analyzed by MALDI TOF-TOF mass spectrometer using a 5800 Proteomics Analyzer. The amino acid sequence was determined *via de novo* sequencing method. PEAKS Studio Version 4.5 SP2 [Bioinformatics Solutions Inc., Waterloo, Canada] was employed to analyze the MS/MS spectra.

The inhibition patterns of the target peptides (F2 and F4) were determined by Lineweaver-Burk plots. Enzymatic assay was carried out using different concentrations (0.6, 1.2, 1.8 and 2.4 mM) of FAPGG as substrate at 37 °C. The inhibition patterns of the peptides were determined in the absence and presence of the inhibitory peptide at two concentrations of 0.06 and 0.12 mg/ml using Lineweaver-Burk plots. Inhibition constant (K_i_) for each peptide was calculated using the secondary plot of ${{\rm{K}}}_{{\rm{m}}}^{{\rm{app}}}$ (i.e. the apparent value of K_m_ at different concentrations of the inhibitor) as a function of the inhibitor concentration. The value of the inhibition constant K_i_ was obtained from the negative x intercept of the secondary plot.

### Molecular modeling and docking

No crystal structure for rabbit ACE enzyme has been reported in Protein Data Bank (PDB). So, the structure of the enzyme was constructed based on the amino acid sequence of rabbit ACE (UniProt ID: P12822) and the crystal structure of human somatic ACE N-domain (PDB ID: 2C6N^17^) as template using MODELLER 9.20^18^. Except for Zn and Cl ions, all other heteroatoms were removed from the original PDB file of 2C6N, and the resulting structure was subjected to restraint-based homology modeling as implemented in MODELLER 9.20^18^. The best model was selected based on the lowest DOPE score and evaluated by PROCHECK^19^, ERRAT^20^ and Verify3D^21^ servers.

In order to predict the binding conformation of IER to ACE enzyme, molecular docking simulation was performed using AutoDock 4.2.5.1^22^. Three-dimensional structure of the IER peptide was built and energy minimized using UCSF Chimera 1.11^23^ by applying Molecular Modelling Toolkit (MMTK) with Amber parameters for standard residues, and 500 steepest descent minimization steps with a step size of 0.02 Å. The molecular structure files were prepared in pdbqt format for both IER and the modeled rabbit ACE using AutoDock Tools. The grid box dimensions were set to 60, 60 and 60 grid points centered on the zinc-binding site and a grid spacing of 0.375 Å was applied.

### Molecular dynamics simulation

Molecular dynamics simulation procedure was conducted using GROMACS 5.0.5^24^ with CHARMM36 forcefiled^25^. The protein-tripeptide complex was solvated in TIP3P water molecules inside a cubic box. Thirteen water molecules were replaced by Na^+^ ions to neutralize the system charge. The neutralized system was then subjected to energy minimization until the maximum force became less than 500 kJ/mol.nm. After energy minimization, two separate position-restrained MD simulations were carried out to equilibrate the system. First, a 200 picoseconds (ps) NVT MD simulation was carried out at 300 K and constant volume using the V-rescale thermostat^26^. Then, Parrinello-Rahman barostat was used for 500 ps to control the system at 1 bar and 300 K^27^. Finally, 100 ns molecular dynamics simulation without position restraint was performed under similar condition. The LINCS algorithm was used to constrain the bonds connecting hydrogen atoms^28^. Long range electrostatic interactions were computed using particle mesh by Ewald method^29^. Both short-range and Lennard-Jones interactions were calculated with a 1.2 nm cut-off distance. A time step of 2 fs was used to integrate the motion equation. The resulting trajectories were analysed using the built-in tools of GROMACS 5.0.5. The minimum energy conformation of the ACE-IER complex was extracted from the trajectory using free energy landscape (FEL) analysis and considered as a representative structure of the complex. The FEL was generated using the gmx sham tool of GROMACS by projecting radius of gyration (Rg) and RMSD to the average structure along with free energy change and visualization of the 2D and 3D diagrams of FEL was done using the trial version of Mathematica 9 (www.wolfram.com/mathematica/trial). The 3D structures were prepared using UCSF Chimera 1.11^23^ and 2D diagrams of ACE-inhibitors were drawn using PoseView (http://proteins.plus)^30^.

## Results and Discussion

### Preparation of protein hydrolysates and ACE inhibition assay

Proteins extracted from *Z. jujuba* fruits were used to purify ACE inhibitory peptides. Bioactive peptides were released by enzymatic digestion using trypsin, papain and a combination of the two. Primary assay showed that all hydrolysates had inhibitory effects on ACE activity. The percentages of ACE inhibitory activity were calculated to be 26.1% (±2.0), 14.2% (±1.2), and 20.8% (±1.8) for trypsin, papain and their combination at a concentration of 0.15 mg/ml, respectively. According to the results, tryptic hydrolysate with the highest ACE inhibitory potential was used for peptide purification.

### Characterizing ACE inhibitory peptides

**Figure 1:**
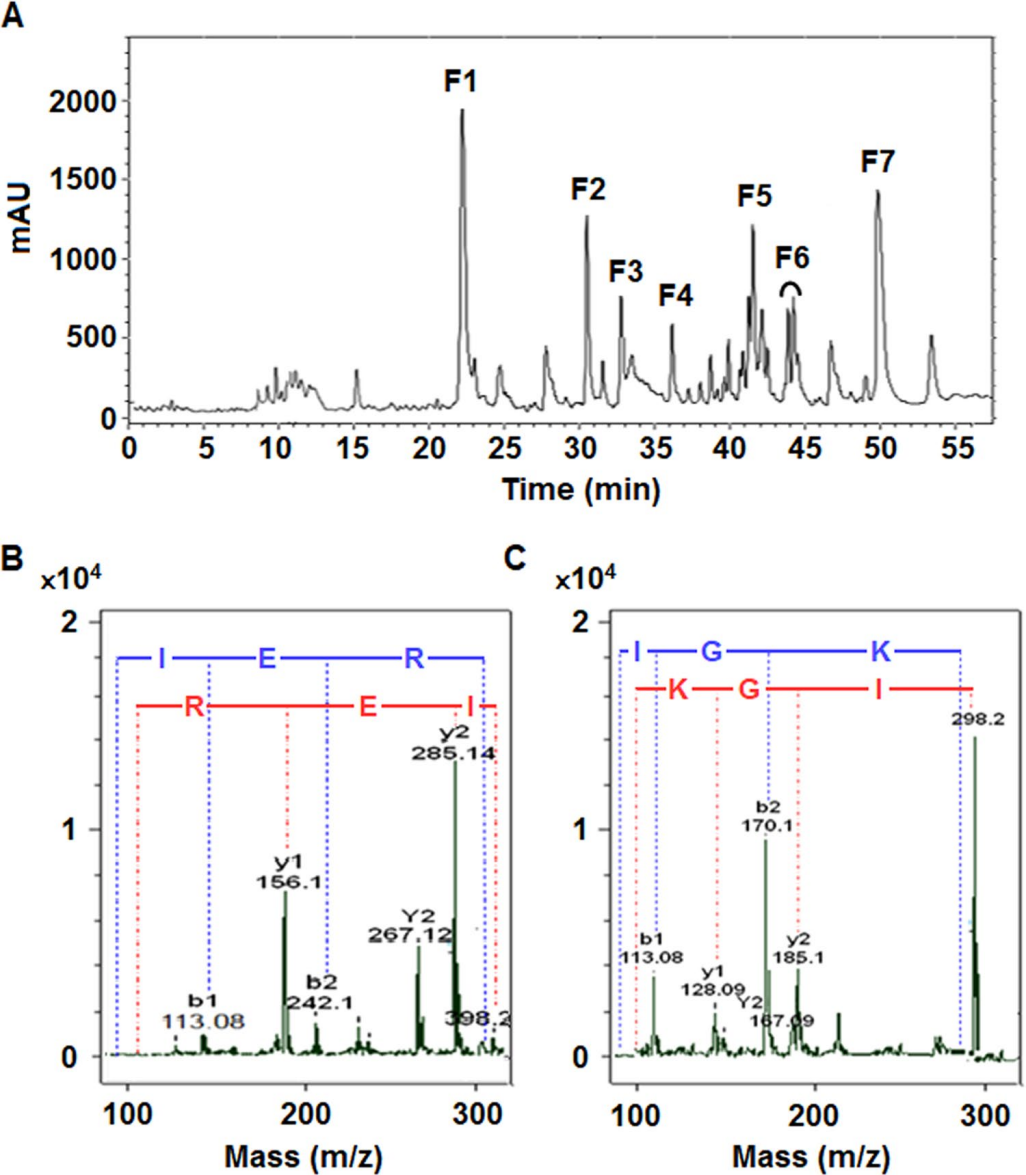
(**A**) HPLC chromatogram of peptides obtained from tryptic hydrolysate of *Z. jujuba* fruit proteins. (**B**,**C**) Identification of the molecular mass and amino acid sequence of the inhibitory peptides using MALDI-TOF (matrix assisted laser desorption/ionization-time of flight). Chromatograms correspond to MS/MS spectra of the F2 and F4 as the most active peptides.

Bioactive peptide fractions were purified from the tryptic hydrolysate using RP-HPLC (Fig. 1A). After ACE inhibitory assay of the major peaks, most active fractions, F2 and F4, were completely purified to obtain a single peak in HPLC chromatogram. Under the same condition, these peptides showed a higher ACE inhibitory activity in comparison with hydrolysate, which is related to the elimination of less effective fractions during peptide purification process. The amino acid sequences of F2 and F4 peptides were identified to be IER and IGK with the IC_50_ values of 0.060 mg/ml (0.144 mM) and 0.072 mg/ml (0.228 mM), respectively (Fig. 1B,C). Accordingly, these small peptides seemed to have higher inhibitory activity towards ACE than some other peptides described in literature as ACE inhibitors including TNLDWY (IC_50_ = 1.932 mM), RADFY (IC_50_ = 1.35 mM), and RVFDGAV (IC_50_ = 1.00 mM) purified from Ginkgo biloba seeds^31^, while they are less active than some other peptides such as KAQYPYV (IC_50_ = 0.0370 mM), KIIIYN (IC_50_ = 0.058 mM), and KILIYG (IC_50_ = 0.053 mM) purified from coconut cake albumin hydrolysates^32^. The IC_50_ value of captopril was calculated to be 2.2 ng/ml which is lower than those of F2 and F4 peptides.

### Determining ACE inhibition pattern

**Figure 2:**
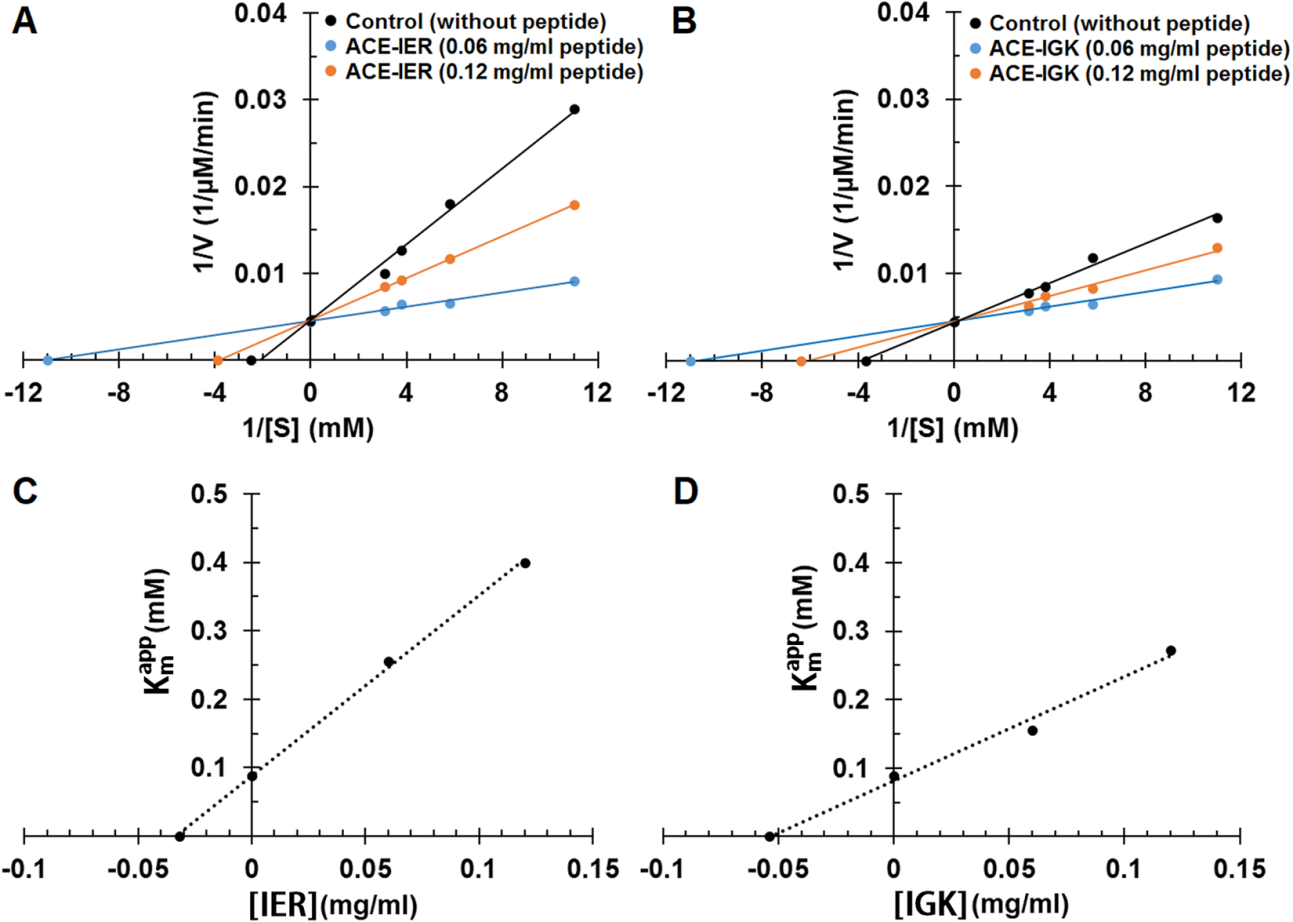
Lineweaver-Burk plots for ACE in the presence and absence of the inhibitory peptides IER (**A**) and IGK (**B**). Secondary plots of as a function of IER (**C**) and IGK (**D**) concentrations. The inhibition constants (Ki) were obtained from the negative x intercepts of the secondary plots.

To determine the ACE inhibition pattern, Lineweaver-Burk plots were generated. Kinetic studies were performed in the absence and presence of 0.06 and 0.12 mg/ml of IER and IGK peptides, separately. V_max_ was found to be unchanged, but K_m_ value was altered, which is an indication of competitive inhibitory pattern (Fig. 2A,B). The K_m_ resulted to be 0.089 mM for the control (without peptide), while it increased to 0.255 and 0.156 mM in the presence of IER and IGK peptides at 0.060 mg/ml of inhibitory peptide, respectively. Furthermore, at 0.12 mg/ml of inhibitory peptide, K_m_ values increased to 0.4 and 0.272 mM for IER and IGK peptides, respectively, which is indicative of reduced affinity of the enzyme for its substrate in the presence of these peptides. Collectively, results imply that these peptides inhibited the enzyme by binding to the free enzyme, but not to the ACE-substrate complex. Since the inhibition pattern of the ACE inhibitory peptides is related to their inhibitory structures, we hypothesize that these peptides may have similar structures to the substrate; hence, they might compete with the substrate for binding to the same interaction site on the enzyme. Most of the ACE inhibitory peptides show competitive inhibition patterns^31^, as is also seen in this study. By using secondary plots (Fig. 2C,D), inhibition constant, K_i_, resulted to be 0.032 and 0.054 mg/ml for IER and IGK peptides, respectively.

### Probing the binding sites by molecular docking

Computational approaches were employed to find out the structural details of the IER binding to the ACE active site. Although the crystal structure of the rabbit ACE enzyme has not been determined so far, it shares a high degree of amino acid sequence identity (88.24% identity for the entire protein sequence and 89.35% identity for the N-domain sequence) with human ACE (UniProt ID: P12821). Hence, the three-dimensional structure of the rabbit ACE was constructed based on the human somatic ACE structure (PDB ID: 2C6N^17^) as the template using MODELLER 9.20^18^. The best modeled structure was selected based on the lowest DOPE score, and the reliability of the predicted structure was validated with PROCHECK, ERRAT and Verify3D servers^19–21^ (Table 1).

**Table 1:** Validation of predicted structure of rabbit ACE.

Server	Feature	Template (2C6N)	Modeled structure^a^	Representative structure^b^
PROCHECK	Residues in most favoured regions (%)	81.6	92.6	94.0
	Residues in allowed regions (%)	17.7	7.2	5.8
	Residues in disallowed regions (%)	0.7	0.2	0.2
Verify3D	Averaged 3D-1D score > 0.2	96.1	92.8	94.7
ERRAT	Overall quality	86.5	87.3	96.5

^a^The structure of the best model in terms of energy, obtained from homology modeling. This structure was used as the starting structure of the MD simulation.

^b^The minimum energy conformation, extracted from the last 90 ns of the MD trajectories using free energy landscape (FEL) analysis.

**Figure 3:**
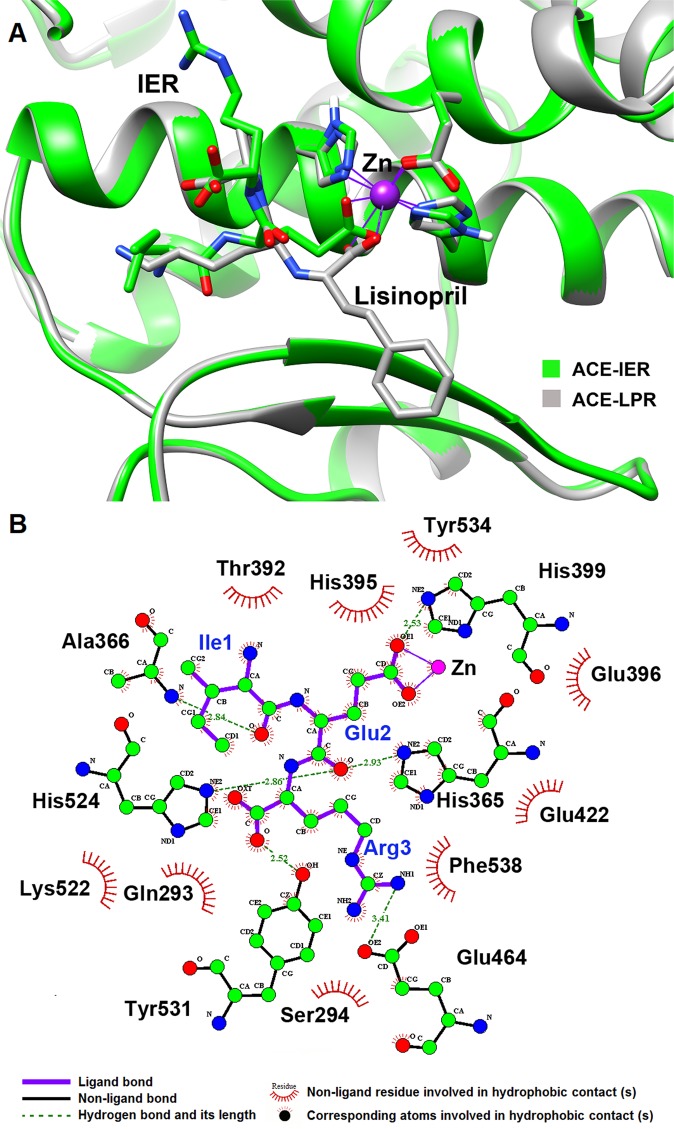
(**A**) Superposition of the three-dimensional structure of ACE-IER complex derived from molecular docking (green) with the crystal structure of ACE in complex with lisinopril obtained from 2C6N pdb entry (gray). Superposition and visualization were done using UCSF Chimera 1.11 (www.cgl.ucsf.edu/chimera). (**B**) 2D diagram of interactions between IER peptide and ACE in the lowest energy conformation of the ACE-IER complex. Purple and black lines represent ligand and non-ligand intramolecular bonds, respectively, green dashed lines present the hydrogen bonds and red dashed half-moons refer to amino acid residues involved in hydrophobic interactions with ligand.

Afterwards, five-hundred docking runs were performed using AutoDock 4.2.5.1^22^ and a conformation of IER showing the lowest binding energy for ACE was extracted for further evaluation. In order to ensure proper binding of IER to the ACE active site, the ACE-IER complex was superimposed on the crystal structure of ACE-lisinopril (PDB ID: 2C6N^17^) which was used as template for homology modeling. Comparison of the structures revealed that the position and orientation of IER peptide in the docked structure were very similar to those of lisinopril in the template crystal structure (Fig. 3A). Analysis of the ACE-IER interaction network using LigPlot^+^ v2.1^33^ indicated that the IER was stabilized in the active site by coordination to zinc ion and six hydrogen bonds involving residues His399, Glu464, Ala366, His365, Tyr531 and His524 (Fig. 3B). The results also implied that IER made additional hydrophobic interactions with Gln293, Ser294, Lys522, Phe538, Glu422, Glu396, Tyr534, His395, Thr392, His399, Glu464, Ala366, His365, Tyr531 and His524.

### Probing the binding site by MD simulation

As mentioned before, there is no crystal structure available for rabbit ACE. So, human ACE enzyme was used as a template to predict the structure of rabbit ACE enzyme by homology modeling. To gain a more realistic insight into the interaction of IER peptide with ACE, the ACE-IER complex was subjected to MD simulation for 100 ns, and the dynamic features of the complex were analyzed.

**Figure 4:**
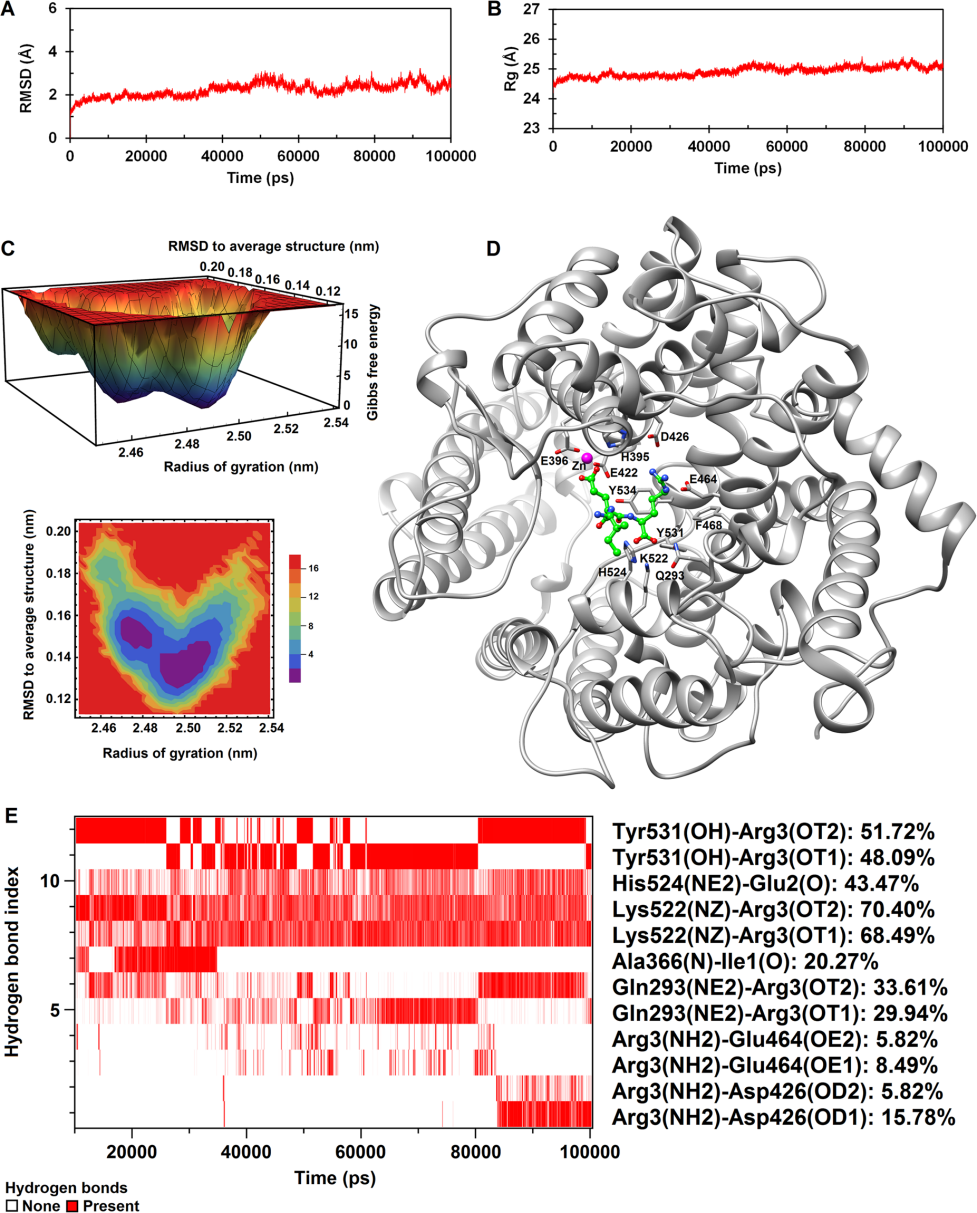
(**A**) RMSD of the carbon alpha (Cα) atoms and (**B**) the radius of gyration of the ACE-IER complex during the entire course of 100 ns MD simulations. (**C**) 3D and 2D plots of free energy landscape (FEL) of the ACE-IER complex as a function of RMSD to the average structure and radius of gyration during the last 90 ns of simulation. The plots were generated using trial version of Mathematica 9 (www.wolfram.com/mathematica/trial). (**D**) 3D visualization of a minimum energy conformation extracted from FEL using UCSF Chimera 1.11 (www.cgl.ucsf.edu/chimera). The interacting residues of ACE with IER are highlighted. (**E**) Map of hydrogen bond existence for ACE-IER complex during the last 90 ns of simulation. Red lines indicate the presence of a hydrogen bond at that specific time. Each value of the hydrogen bond index corresponds to a unique donor-hydrogen-acceptor triplet. The amino acids involved in each particular hydrogen bond and the percentage of time that the hydrogen bond existed during the trajectory are given on the right side of the map.

Time evaluation of root mean square deviation (RMSD) for the alpha carbon (Cα) atoms and gyration radius of the protein are shown in Fig. 4A,B. As seen, after 10 ns, ACE-IER complex reaches a relatively stable conformation. Accordingly, the last 90 ns of the simulation time were chosen for further analysis. Free energy landscape analysis was used to extract a representative conformation with the lowest free energy from the trajectories of the last 90 ns of MD simulation (Fig. 4C,D). Calculating the probability of hydrogen bonds existence revealed more details on the residues involved in the IER binding to the enzyme active site (Fig. 4E). Throughout the simulation, four hydrogen bonds between IER and ACE within the docked structure were broken or significantly weakened, but a new network of hydrogen bonds was formed which stabilized the IER in its location within the ACE active site.

The hydrogen bonds with existence probability greater than 5% are depicted in Fig. 4E. As seen, the IER peptide was hydrogen bonded to the residues Gln293, Ala366, Asp426, Glu464, Lys522, His524, and Tyr531. Among these, the Lys522-Arg3, His524-Glu2, Tyr531-Arg3 and Gln293-Arg3 hydrogen bonds existed persistently throughout the simulation time. Hydrogen bond partners of Tyr531 and Gln293 were alternatively switched between oxygen atoms OT1 and OT2 of Arg3. Two hydrogen bonds Ala366-Ile1 and Arg3-Glu464 were broken and weaken, respectively. In addition, Asp426 established a relatively strong hydrogen bond with Arg3 during the last 15 ns of the simulation (Fig. 4E). Analysis of the contacts within a radius of 5 Å revealed that the residues Tyr531, Lys522, His524, Glu396, His395, Gln293, Phe468, Tyr534 and Glu422 were involved in hydrophobic interactions with IER peptide (Table 2).

**Table 2:** The contacts between ACE and IER (within radius of 5 Å) with occupancy greater than 50% during the last 90 ns of simulation.

Residue of ACE	Residue of IER	Occupancy (%)	Residue of ACE	Residue of IER	Occupancy (%)
Tyr531 (HH)	Arg3 (C)	100.00	His524 (NE2)	Arg3 (HA)	67.19
Lys522 (NZ)	Arg3 (OT2)	98.22	Phe468 (HE1)	Arg3 (HB2)	66.22
Lys522 (NZ)	Arg3 (OT1)	97.45	Tyr531 (OH)	Arg3 (HB2)	65.32
His524 (HE2)	Glu2 (O)	96.89	His524 (HE2)	Glu2 (C)	64.73
Lys522 (NZ)	Arg3 (C)	95.36	Tyr534 (CD1)	Arg3 (HG1)	64.42
His524 (HE1)	Arg3 (C)	91.52	His524 (CE1)	Arg3 (HA)	64.13
His524 (NE2)	Glu2 (O)	90.62	Gln293 (2HE2)	Arg3 (HB2)	63.12
Glu396 (OE2)	Glu2 (OE2)	89.45	Glu396 (OE2)	Glu2 (OE1)	62.52
Lys522 (HZ3)	Arg3 (OT2)	89.01	His524 (CE1)	Glu2 (O)	62.44
His395 (NE2)	Glu2 (OE2)	87.27	Tyr531 (HH)	Arg3 (CA)	62.41
His524 (HE1)	Arg3 (HA)	86.29	Tyr531 (HE1)	Arg3 (C)	60.09
His524 (HE2)	Arg3 (HA)	85.79	Glu422 (OE2)	Glu2 (OE2)	59.11
Lys522 (HZ2)	Arg3 (OT1)	85.48	Tyr531 (HH)	Arg3 (OT2)	57.33
Lys522 (HZ3)	Arg3 (OT1)	84.69	Lys522 (HZ3)	Arg3 (C)	55.81
Tyr531 (HH)	Arg3 (HB2)	84.37	Glu396 (OE2)	Glu2 (CD)	55.37
Tyr531 (HE1)	Arg3 (HA)	83.48	Tyr534 (HB2)	Arg3 (HG1)	54.69
Lys522 (HZ2)	Arg3 (OT2)	82.60	Tyr534 (HB2)	Arg3 (HD1)	54.48
His524 (HE1)	Glu2 (O)	81.92	Lys522 (CE)	Arg3 (OT2)	53.94
Lys522 (HZ1)	Arg3 (OT1)	80.14	Tyr534 (HD1)	Arg3 (HG1)	53.77
His524 (HE1)	Arg3 (OT1)	78.82	Tyr531 (HH)	Arg3 (OT1)	53.24
Lys522 (HZ1)	Arg3 (OT2)	78.69	Tyr531 (HE1)	Arg3 (OT2)	52.92
His524 (HE1)	Arg3 (OT2)	77.10	Lys522 (HE2)	Arg3 (OT2)	52.68
Gln293 (2HE2)	Arg3 (C)	73.52	Tyr531 (OH)	Arg3 (OT2)	52.02
His524 (HE1)	Arg3 (CA)	70.74	Tyr534 (CG)	Arg3 (HG1)	51.59
Tyr531 (HH)	Arg3 (HA)	70.41	Tyr534 (HH)	Glu2 (HB2)	50.16
Gln293 (2HE2)	Arg3 (OT1)	68.54	Tyr534 (HB2)	Arg3 (HD2)	50.05
Gln293 (2HE2)	Arg3 (OT2)	68.19			

**Figure 5:**
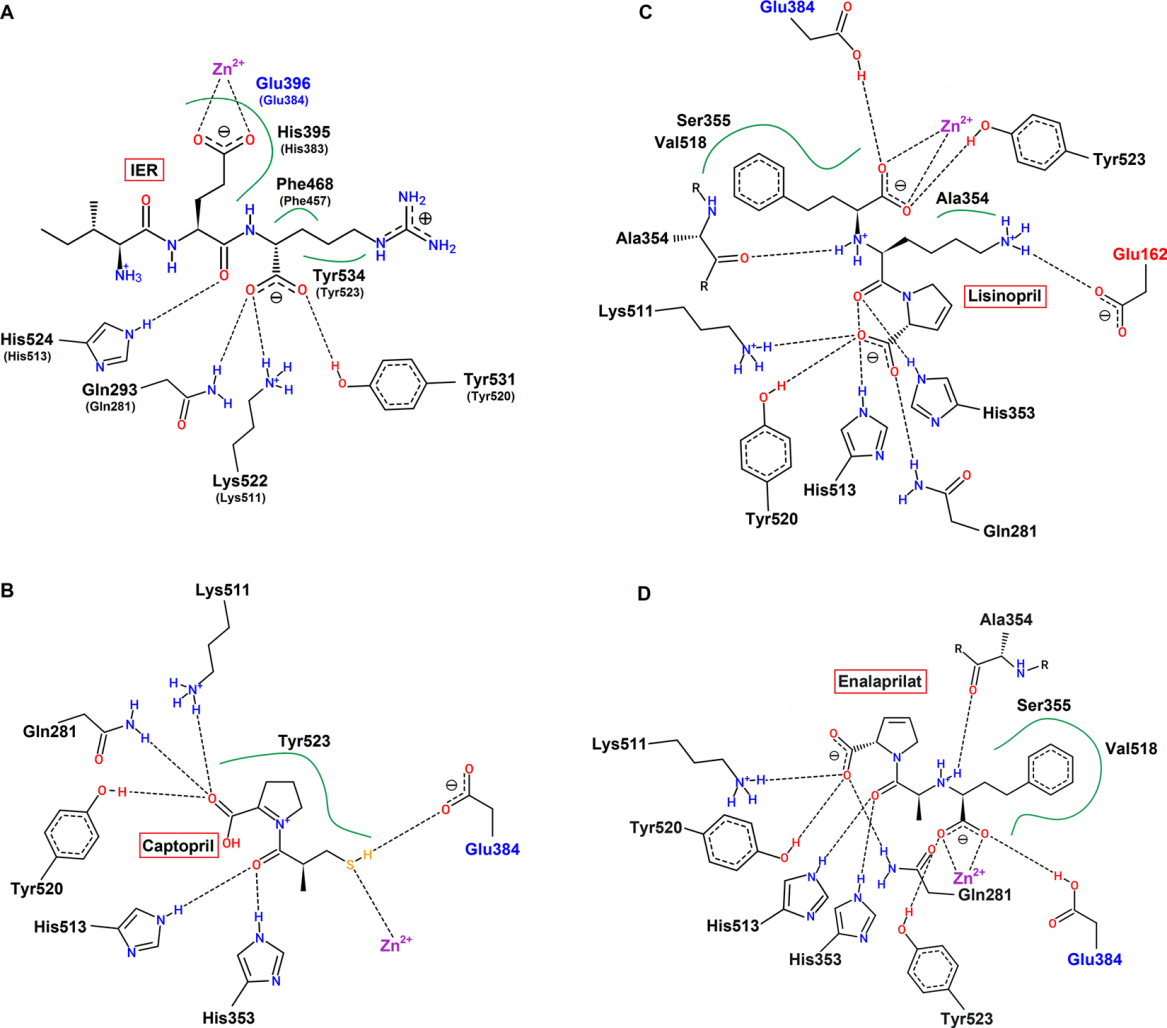
A schematic representation of interaction network of IER, captopril, lisinopril and enalaprilat with ACE active site generated with PoseView (http://proteins.plus). Residues of S1, S1’ and S2’ subsites are marked in blue, red and black, respectively and Zn^2+^ is shown in purple. The hydrophobic interactions are highlighted with green lines. (**A**) IER forms hydrogen bonds with Gln293, Lys522, His524 and Tyr531 of rabbit ACE. The complex is also stabilized by the hydrophobic interactions with Glu396, His395, Phe468 and Tyr534. The equivalent residues in the human testicular ACE are enclosed in parentheses. (**B**) Captopril makes hydrogen bonds with residues Gln281, His353, Glu384, Lys511, His513 and Tyr520 of human testicular ACE and interacts with Tyr523 through hydrophobic contact. (**C**,**D**) Lisinopril and enalaprilat participate in hydrogen bonding with residues Gln281, His353, Ala354, Glu384, Lys511, His513, Tyr520 and Tyr523. The lisinopril also occupies S1’ subsite through hydrogen bonding between lysine side chain and Glu162. The phenyl moiety of these inhibitors which is absent in captopril mediates hydrophobic interactions with residues Ser355 and Val518.

Structural comparison of the interaction networks of IER, captopril, enalaprilat and lisinopril with ACE active site indicates that the results of this study are in good agreement with those obtained for known competitive inhibitors of testicular ACE^34,35^. A more detailed look at the IER binding site revealed that the arginine residue of IER peptide makes hydrogen bonds with Gln293, Lys522 and Tyr531 and also hydrophobic interactions with Phe468 and Tyr534 of ACE. The glutamate residue of IER, which is coordinated to Zn^2+^, makes a hydrogen bond with His524 via its backbone oxygen and also interacts with Glu396 and His395 through hydrophobic contacts (Fig. 5A). The residues Gln293, His395, Glu396, Phe468, Lys522, His524, Tyr531 and Tyr 534 are equivalent to Gln281, His383, Glu384, Phe457, Lys511, His513, Tyr520 and Tyr523 of human testicular ACE, respectively. Structural analysis of the ACE inhibitors shows that captopril, which is a small thiol-containing compound, forms hydrogen bonds with Glu384 (S1 subsite), Gln281, His353, Lys511, His513 and Tyr520 (S2’ subsite) of human testicular ACE^34^ (Fig. 5B), while bulkier inhibitors lisinopril and enalaprilat establish two more intermolecular hydrogen bonds with Ala354 and Tyr523 residues and their phenyl moiety (absent in captopril) contributes to hydrophobic interaction with Ser355 and Val518 (Fig. 5C,D). In addition, lisinopril which is a lysine derivative of enalaprilat, can also interact with Glu162 from the deep S1’ subsite due to its long lysine side chain (Fig. 5C). The other two inhibitors have a small methyl group instead of lysine. Although the IER contains an isoleucine in this position which is more bulky than the methyl group, but still is not able to establish any interaction with deep S1’ subsite, probably due to the lack of a positively charged group and only occupies S1 and S2’ subsites of ACE in a very similar way as captopril and enalaprilat. Taken together, it seems that IER inhibits ACE in a competitive manner, as supported by experimental and structural evidences.

## Conclusion

In this study, we purified and characterized two tripeptides, IER and IGK, that have the ability to inhibit ACE with the IC_50_ values equal to 0.060 and 0.072 mg/ml, respectively. Analysing the inhibition mechanism by Lineweaver-Burk plots revealed that these peptides compete with the substrate in binding to the enzyme active site. MD simulation results implied that the IER peptide, similar to common inhibitors of ACE such as captopril, binds to the active site and occupies the S1 and S2’ subsites of ACE, confirming that the IER peptide inhibits the rabbit ACE enzyme through a competitive mechanism.
